# Tumor Immunogenic Cell Death as a Mediator of Intratumor CD8 T-Cell Recruitment

**DOI:** 10.3390/cells11223672

**Published:** 2022-11-18

**Authors:** Nicolas Roussot, François Ghiringhelli, Cédric Rébé

**Affiliations:** 1 Cancer Biology Transfer Platform, Centre Georges-François Leclerc, F-21000 Dijon, France; 2 Equipe Labellisée Ligue Contre le Cancer, Centre de Recherche INSERM LNC-UMR1231, F-21000 Dijon, France; 3 UFR Sciences de Santé, University Bourgogne Franche-Comté, F-21000 Dijon, France; 4 Department of Medical Oncology, Centre Georges-François Leclerc, F-21000 Dijon, France; 5 Genetic and Immunology Medical Institute, F-21000 Dijon, France

**Keywords:** immunogenic cell death, CD8, chemokines, immune checkpoint blockers

## Abstract

The success of anticancer treatments relies on a long-term response which can be mediated by the immune system. Thus, the concept of immunogenic cell death (ICD) describes the capacity of dying cancer cells, under chemotherapy or physical stress, to express or release danger-associated molecular patterns (DAMPs). These DAMPs are essential to activate dendritic cells (DCs) and to stimulate an antigen presentation to CD8 cytotoxic cells. Then, activated CD8 T cells exert their antitumor effects through cytotoxic molecules, an effect which is transitory due to the establishment of a feedback loop leading to T-cell exhaustion. This phenomenon can be reversed using immune checkpoint blockers (ICBs), such as anti-PD-1, PD-L1 or CTLA-4 Abs. However, the blockade of these checkpoints is efficient only if the CD8 T cells are recruited within the tumor. The CD8 T-cell chemoattraction is mediated by chemokines. Hence, an important question is whether the ICD can not only influence the DC activation and resulting CD8 T-cell activation but can also favor the chemokine production at the tumor site, thus triggering their recruitment. This is the aim of this review, in which we will decipher the role of some chemokines (and their specific receptors), shown to be released during ICD, on the CD8 T-cell recruitment and antitumor response. We will also analyze the clinical applications of these chemokines as predictive or prognostic markers or as new targets which should be used to improve patients’ response.

## 1. Introduction

Over the past decade, the most effective therapies used to treat cancers rely on the antitumor immune response. This effectiveness is widely due to a marked and durable response. The optimal antitumor immune response is driven by the presentation of tumor antigens by APCs (antigen-presenting cells) to CD8^+^ cytotoxic T cells. These lymphocytes exert their action on tumor cells by secreting cytotoxic molecules, such as granzyme B, perforin or IFNγ, to maintain antitumor immunity. While the tumor antigen presentation was shown to be improved by immunogenic tumor cell death (ICD) inducers (mainly chemotherapeutic agents), the sustained CD8 T-cell activity can be triggered by immune checkpoint blockers (ICBs: anti-PD-1/PD-L1 and anti-CTLA-4 Ab). After antigen stimulation, CD8 T cells progressively lose their effector functions and overexpress PD-1. Blocking the PD-1/PD-L1 pathway reinvigorates the CD8 T cells, thus driving their proliferation and the restoration of their functions in humans and mice [1].

However, the blockade of the checkpoints will be efficient only if CD8 T cells are recruited within the tumor. This recruitment is heterogeneous, even for tumors from the same organ, and may depend on the tumor mutation status, the tumor microenvironment (TME) or treatments. The CD8 T-cell infiltration was proposed as a marker to identify poorly infiltrated non-responders to chemo-immunotherapy “cold tumors” as compared to “hot tumors”, highly infiltrated and responders [2].

The CD8 T-cell chemoattraction is mediated by chemokine gradients from the starting initial localization of CD8 T cells to the arrival scene, where chemokines are produced. The four main classes of chemokines are established according to the location of the first two cysteine (C) residues in their protein sequence: the CC, CXC, C and CX3C chemokines. Chemokines able to chemoattract CD8 T cells will be the ligands of receptors expressed at their cell surface. Most chemokine receptors are transmembrane-spanning heterotrimeric G-protein-coupled receptors. The binding of chemokines on their receptors induces the G-protein coupling and the subsequent activation of the downstream signaling proteins involved in cell migration, such as Rac, Rho and Cdc42, thus leading to the moving of the cells toward the chemotactic gradient [3].

The main receptors expressed on the CD8 T-cell membrane are CXCR3, CXCR4, CXCR6, CCR2, CCR5, CCR6 and CX3CR1 [4,5,6]. Each of them recognizes one or several chemokines. The main receptor/ligands implicated in this process are compiled in Table 1.

The aim of this review is to understand how immunogenic cell death can influence the recruitment of CD8 T cells at the tumor site to improve the antitumor immune response.

## 2. Immunogenic Cell Death (ICD)

**Figure 1:**
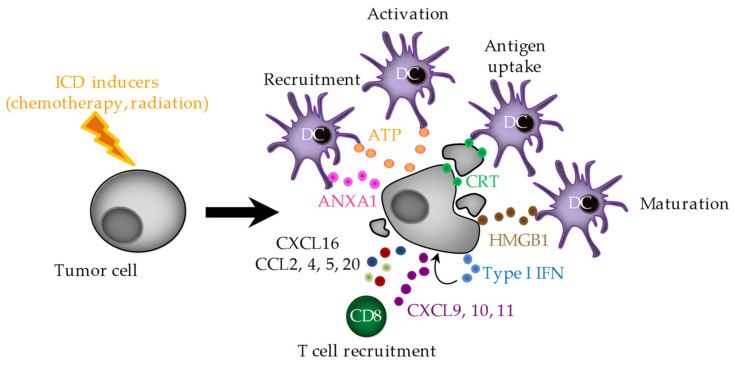
Immunogenic cell death (ICD) features. ICD is characterized by ER stress and calreticulin (CRT) exposure, ATP, HMGB1 (High-Mobility Group Box 1), ANXA1 (annexin A1) and type I interferon (IFN) production by dying cancer cells, thus leading to DC recruitment, maturation and activation. ICD is also characterized by chemokine production (mediated in some circumstances by type I IFN) which in turn triggers CD8 T-cell recruitment at the tumor site.

One of the first steps to start the antitumor immune response is the tumor antigen recognition by the APCs. This phase can be sensitized by the DAMPs (danger-associated molecular patterns) which are expressed at the cell surface or released by dying cancer cells. Thus, some chemotherapeutic drugs or physical treatments (such as irradiation) have the capacity to kill cancer cells through ICD, a type of cell death identified to activate an adaptive immune response specific to tumor antigens. ICD is mainly characterized by calreticulin (CRT) exposure at the cell surface of dying tumor cells, the release of the ATP, HMGB1 (High-Mobility Group Box 1) and annexin A1 (ANXA1) in the extracellular space. Finally, ICD is characterized by the activation of an intrinsic type I interferons (IFNs) pathway which triggers the CXCL10 release by dying cancer cells in an autocrine signaling loop (Figure 1) [22].

Chemotherapeutic and physical treatments were shown to be able to induce all or part of these features, depending on the cell type, concentration and time of exposure. For example, some platinum derivatives were shown to be able to induce ICD features in some studies but not in other ones [23]. Such a discrepancy might rely on the tumor type which could influence the apparition of the ICD process.

### 2.1. ER Stress and CRT Exposure

CRT exposure at the cell surface of dying cells will behave as an “eat me” signal for dendritic cells (DCs). CRT is a chaperon protein that fixes calcium at the ER level [24]. Three steps are required to lead to CRT exposure. (1) ER stress with eukaryotic Initiation Factor 2α (eIF2α) phosphorylation. (2) An apoptotic signal with caspase-8 activation and consequently the cleavage of its target Bap31. The cleaved Bap31 entails the Bax and Bak oligomerization, leading to the disruption of the mitochondrial permeability and the cytochrome c release in the cytosol. (3) A transport from the ER to the Golgi apparatus engaged by the two first steps, which allows CRT exocytosis through its translocation from the ER to the plasma membrane. This transport is dependent on VAMP1 (Vesicle-Associated Membrane Protein 1) and SNAP25 (SyNaptosomal-Associated Protein 25) [25]. Then, the membrane-associated CRT is recognized by the APCs (DCs, macrophages and neutrophils) through CD91 and CD69 and favors the phagocytosis of apoptotic bodies [26].

### 2.2. ATP Release

The ATP release from dying tumor cells can be induced by several mechanisms. However, the main pathway is autophagy which enables high levels of intracellular ATP. The ATP included in cytoplasmic vesicles is transferred into autolysosomes through an autophagy process dependent on ATG5 (autophagy-related genes 5), ATG7 and Beclin1. Autolysosomal LAMP1 (Lysosomal-Associated Membrane Protein 1) favors the lysosome membrane rupture and ATP release in the cytosol or in the extracellular environment when autolysosomes merge with the plasma membrane. ATP can also be released outside of the cell by the membrane channel pannexin 1 after activation by caspases [27,28]. Extracellular ATP plays an attractant role at the tumor site on immune cells, through the fixation on its receptor P2Y2 expressed by monocytes or DCs. Once recruited and activated by the “eat me” signals in the tumor, naïve immune cells need further activation signals. ATP signaling via P2RX7 leads to the release of potassium ions and to the NLRP3 inflammasome activation in DCs and macrophages. The assembly of the NLRP3 inflammasome triggers the caspase-1 activation, which in turn cleaves and maturates IL-1β and IL-18, two pro-inflammatory cytokines [29]. Although P2RX7 is expressed on many cell types, ATP seems to act mainly on DCs after ICD.

### 2.3. HMGB1 Release

HMGB1 is a ubiquitous protein playing an important role in nucleosome stability, transcription regulation and DNA repair. In addition to its nuclear function, HMGB1 plays a role in inflammation, cell differentiation and migration and the tumor metastasis of the remaining cells after chemotherapy [30]. HMGB1 is released by dying tumor cells after nuclear and cytoplasmic membrane permeabilization. HMGB1 binds Toll-like receptors (TLR) 2 and 4 or RAGE (receptor for advanced glycation end products) [31,32]. However, the binding of HMGB1 to DC TLR4 might be the signal driving the perception of the ICD by restricting the lysosomal degradation of the phagocyted material, then leading to the efficient processing and cross-presentation of dying tumor-derived antigens by DCs. Thus, cell death cannot be sensed as immunogenic in the absence of HMGB1 in cancer cells or TLR4 in myeloid cells [33].

### 2.4. ANXA1 Release

ANXA1 has the role of the homing factor to recruit DCs or their precursors to cancer cells undergoing ICD, through binding to its receptor formyl peptide receptor 1 (FPR1). Thus, when cancer cells lack *Anxa1* or when the host is deficient for *Fpr1*, the anticancer activity of anthracyclines in mice is compromised. Moreover, the loss-of-function polymorphisms in *FPR1* are associated with poor overall survival and metastasis-free survival in breast cancer patients treated with adjuvant anthracycline-based chemotherapy [34].

### 2.5. Type I IFNs

The type I IFN response is triggered by both RNA and DNA species. While RNA is recognized by endosomal TLR3, DNA is sensed by cGAS (cytosolic cyclic GMP-AMP (cGAMP) synthase) and STING (stimulator of IFN response cGAMP interactor 1) [35,36]. Once secreted, type I IFN binds to the heterodimeric IFNAR1/2 (IFNα receptor 1/2) expressed on various immune cells, thus leading to immunostimulatory events, such as the enhancement of CD8 T- and NK-cell cytotoxic functions [37] or cross-priming by DCs [38].

### 2.6. Chemokines

Besides these direct functions on immune cells, dying cancer cell-derived type I IFN also triggers the synthesis of CXCL10 via an autocrine signaling loop [35]. Moreover, the CXCL10 production can also be induced after the engulfment of chemotherapy-damaged mitochondria in autophagolysosomes and the sensing of released mitochondrial DNA by TLR9 [39]. Thus, ICD inducers are able to induce the CXCL10 secretion by cancer cells, raising CXCL10 as another ICD feature. For example, oxaliplatin induces the CXCL10 production by melanoma cells [40] and cisplatin triggers the CXCL10 (together with CRT) expression in vivo in a B16 melanoma model [41]. While the production of CXCL10 was not investigated in CT26 or LL/2 tumor-bearing mice treated with cisplatin, the addition of the recombinant CXCL10 increases the antitumor effects [42]. Moreover, the immunogenic dying cancer cells were proposed to trigger a pathogen response-like chemokine signature, the CXCL1, CCL2 and CXCL10 co-release. This signature is responsible for the recruitment of neutrophils as the first innate immune responders [43].

Kroemer’s team also showed that some ICD inducers were able to induce the expression of a lot of chemokines within tumors, such as CCL11, 17, 22 and CXCL16 (induced by oxaliplatine) or CCL1, 2, 4, 5, 9, CXCL2, 9, 10, 11, 12, 13 and 14 (oxaliplatine and mithoxanthrone). Among these chemokines, some of them seemed not to be induced by cisplatin (CCL1, 2, 4, 5, 9, 11, 17, 22 or CXCL2 and 13), a non-inducer of ICD in this context, suggesting that their expression would be driven only by ICD inducers [44]. The association of the chemokine release with ICD is strengthened by the observation that CXCL2 (or its human ortholog CXCL8) is responsible for the translocation of the CRT to the outer leaflet of the plasma membrane [44].

Then, other studies have highlighted the production of chemokines during ICD. In bladder cancer cell lines, mitomycin C was shown to induce the expression of CXCL2, 3, 5, 6 and CCL20 [45]. Radiations which are known to induce ICD stigmata also induce CXCL16 in human breast cancer cells and in diverse murine cancer cell lines [46,47]. Along the paclitaxel-mediated ICD, the cancer cell autonomous TLR4 signaling is essential to the release of DAMPs, which led, in an autocrine manner, to the activation of the NF-κB-mediated CCL2 transcription, together with CXCL10 [48]. Moreover, crizotinib, an FDA-approved TKI able to allow cisplatin to induce ICD, was also shown to induce *Cxcl5* expression within tumors in association with cisplatin [49]. The cyclin-dependent kinase inhibitor dinaciclib, which is able to induce ICD, also triggers the expression of type I IFN and MHC class I gene expression and *Ccl5*, *Cxcl10* and *Ccl2* [50]. In human NSCLC cells, docetaxel induces the release of HMGB1 which in turn (such as recombinant HMGB1) is able to induce the CXCL11 expression and production [51]. Another role of HMGB1 was shown by its capacity to bind to CXCL12, thus allowing DCs’ chemotaxis via CXCR4 signaling [52].

## 3. CD8 T-Cell Chemoattraction by Chemokines Released during ICD

**Figure 2:**
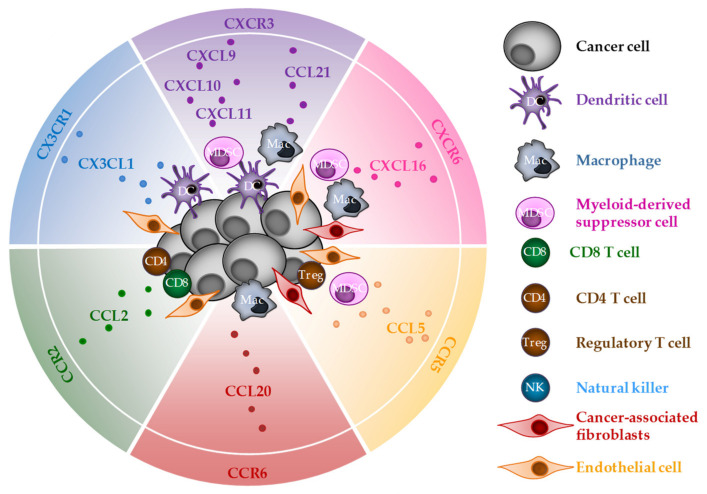
Chemokines and chemokine receptors that may be involved in CD8 T-cell chemoattraction during ICD. Cells in the tumor microenvironment and cancer cells (at the center) able to secrete chemokines and corresponding receptors (at the periphery) expressed by CD8 T cells.

One of the features of ICD is the chemokine production in tumors. Once released, these molecular actors will attract cells expressing specific receptors. Thus, one chemokine will regulate the migration of many cell types with potent pro- or antitumor effects. Here, we will focus on CD8 T cells to decipher how ICD inducers may improve the CD8 T-cell-dependent antitumor immune response. Among the chemokines described to be released during ICD, some reports identified several chemokine/receptor couples able to regulate the CD8 T-cell migration: CXCL9, 10, 11/CXCR3, CXCL16/CXCR6, CX3CL1/CX3CR1, CCL2/CCR2, CCL3, 4, 5/CCR5 and CCL20/CCR6 (Figure 2).

### 3.1. CXCR3

CXCR3 is expressed by CD8 T cells but can also be found at the membrane of CD4 Th1, NK cells and macrophages. It is the receptor for the Th1-type chemokines CXCL9, CXCL10 and CXCL11. When these molecules are highly produced within the tumor, their ligation with CXCR3 allows their migration into tumors. This phenomenon was observed in different murine cancer models, such as breast or renal cancer, lymphoma or melanoma [7,8,11,12,53]. Different types of cells forming the tumor microenvironment were shown to be able to produce CXCR3 ligands, such as myeloid cells (DCs, macrophages or myeloid-derived suppressor cells (MDSCs)) and cancer cells themselves [35,39,54,55,56,57].

In a colon cancer model with a heterogeneous response to avelumab (anti-PD-L1), single-cell RNA sequencing on biopsies before treatment allowed the discovery of a *Cxcl9*^+^ macrophage subset which is associated with the response. Furthermore, blocking CXCL9 in mice canceled the tumor growth inhibition initially obtained with avelumab [58].

When melanoma or ovarian cancer cells overexpressing CXCL9 are s.c. injected into mice, the CD4 and CD8 T-cell recruitment and activation are increased and the tumor growth is slowed down [59,60]. Similarly, the CXCL9/10/11 overexpression in Lewis Lung carcinoma cells increases the CD8 T-cell recruitment and controls the tumor growth alone or in association with an anti-PD-1 Ab [61].

In the same way, several approaches were tested to deliver DNA coding for CXCR3 ligands in the tumors. Thus, the electroporation of established colon tumors with a plasmid coding for CXCL9 together with IL-12 increases the CD8 T-cell recruitment and delays the tumor growth, either alone or in combination with an anti-PD-1 Ab [62]. CXCL11-armed oncolytic poxvirus i.p. injected into mesothelioma-bearing mice favors the CD8 T-cell accumulation in the tumor which is then responsible for an efficient antitumor immune response [63]. In a colon carcinoma model, the intratumor injection of an adenovirus armed with the *Cxcl10* gene allows the recruitment of CD8 T cells. In this context, an anti-PD-1 Ab must be added to have an antitumor effect [64].

Besides overexpression, another approach is to find chemokine inductors. Intratumor injections of the STING agonist were shown to activate the type I IFN pathway, thus leading to chemokine expression (CXCL10, CCL5 and CXCL2), CD8 T-cell recruitment in a CXCR3-dependent manner and pancreatic tumor growth delay [65]. Moreover, the use of romidepsin, an HDAC inhibitor, or celecoxib, a COX2 inhibitor, reinforces the expression of chemokines such as CXCL9/10 or CCL5 in cancer cells, macrophages and T cells. This allows the recruitment of CD8 T cells in melanoma and lung tumors and the slowdown of tumor growth, both improved when using an anti-PD-1 Ab [66,67]. Similar results were obtained with entolimod or imiquimod, two agonists of TLR5 and TLR7, respectively, which triggered a CD8 T-cell chemoattraction through an INFγ-CXCL9/10 pathway and colon or vaginal tumor growth delay [68,69]. In murine hepatocellular carcinoma models, regorafenib—a multikinase anti-VEGFR—combined with anti-PD1 confers a survival benefit which requires the presence of CD8 T cells. Indeed, CXCL10 expression is enhanced by the combination therapy, thus promoting CXCR3-mediated cytotoxic T-lymphocyte infiltration and leading to a delay in hepatocarcinoma tumor growth [70]. We also showed the importance of CXCL10 in the chemo-immunotherapy response. In lung cancer models mutated for KRAS, the overactivation of MEK-ERK blocks the CXCL10 expression in cancer cells. Thus, inhibiting this pathway with trametinib allows chemotherapy (cisplatin/pemetrexed) to increase the CXCL10 expression and production by cancer cells, the recruitment of CD8 T cells within the tumor and the control of the tumor growth when associated with the anti-PD-1 antibody [39]. Finally, a single injection of cyclophosphamide, a drug able to induce ICD, can increase the *Cxcl9*, *Cxcl10* and *Cxcl11* expression within mastocytoma tumors (an effect dependent on CD4 T cells), thus allowing the recruitment of CD8 T cells and tumor regression [71]. Moreover, the association of cyclophosphamide with a TLR9 agonist increases the chemokine expression in murine colon tumors (*Ccl5*, *Cxcl9* and *Cxcl10*) and CD8 T-cell proportion and inhibits tumor growth [72].

Another way to increase chemokine expression is to remove transcription inhibition, such as promoter methylation. In this setting, the use of EZH2-mediated histone modification (GSK126) and DNA-methylation (5-AZA dC) inhibitors removes the *Cxcl9* and *Cxcl10* transcription repression in ovarian cancer cells, thus increasing the CD8 T-cell recruitment in the tumor, slowing down the tumor growth and improving the anti-PD-L1 therapy efficacy [73]. Similarly, the methyltransferase inhibitor decitabine increases the *Cxcl10* (and also other chemokines, such as *Ccl5*) expression by ovarian cancer cells. This increased chemokine expression is associated with improved CD8 and NK cell numbers in the tumors and an improved response to the anti-CTLA-4 Ab [74].

Other strategies have been evaluated. First, the inhibition of the dipeptidylpeptidase DPP4 dampens the CXCL10 degradation, thus improving the CD4 and CD8 T-cell recruitment and limiting the melanoma and colon tumor growth in the presence or not of anti-PD-1 and/or anti-CTLA-4 [75]. Second, an immunotherapeutic cocktail comprising a vaccine, chemotherapy and a TLR3 agonist (VCT) specific for hardly curable tumors was administrated to melanoma and glioma tumor-bearing mice. It induces high amounts of CCL5 and CXCL10 in tumor cells with distinct kinetics. In this context, CXCL10 was shown to contribute to the therapeutic success of this treatment by promoting the recruitment of CXCR3^+^CD8^+^ T cells at the tumor site. However, the secretion of CCL5 by cancer cells drives the accumulation of immunosuppressive CCR5^+^ cells and not CD8 T cells in this context. Thus, the blockade of CCL5 improves the therapeutic efficacy of the VCT [76]. The importance of these two chemokines was also shown in deficient DNA mismatch repair colorectal cancer (CRC). Due to their unstable genomes, these cells produce particular cytoplasmic DNA fragments (at baseline or under chemotherapeutic treatments) which sense the cGAS/STING and type I IFN signaling pathways to induce the production of CXCL10 and CCL5 and the recruitment of CD8 T cells at the tumor site. However, the impact of this pathway on tumor growth was not evaluated in this study [77]. A similar observation was made in multiple myeloma models. Bortezomide is able to induce ICD in these models and especially type I IFN and CXCL9 production in a STING-dependent manner which is associated with an increase in the lymphocytes at the tumor site. Moreover, bortezomide slows down multiple myeloma tumor growth, an effect emphasized by a STING agonist [78].

Another way to increase the chemokine expression within the tumors is to use cellular therapy. MSC (mesenchymal stem cells) are promising cellular vehicles that possess an intrinsic preferential migratory ability toward a number of different tumor types upon systemic administration. Hence, using MSC engineering to deliver CXCL9 (and also immunostimulatory factors OX40L and TNFSF4) triggers an increase in the CD8 and NK cells expressing granzyme B in colon tumor models. In this therapeutic context, both the CXCL9 and immunostimulatory factors are required to improve the antitumor response, an effect which is upgraded in association with an anti-PD-1 Ab [79].

Moreover, an amplification loop seems to exist between CXCL9, 10 and/or 11 and ICBs, as anti-PD-1 or anti CTLA-4 Abs allow an increase in these chemokines within murine and human tumors [56,57,80]. Thus, the upregulation of CXCL9 and 10 by ICBs enhances T-cell homing in a CXCR3-dependent manner. Interestingly it appears that the main source of CXCL9 is macrophages in multiple tumor models [57]. These data emphasize the role of other immune cells in the tumor microenvironment that are critical for ICB efficacy.

Because the number of activated CD8 T cells within melanoma or colon tumor models is decreased in Cxcr3^−/−^ mice, some studies aimed at identifying the role of this pathway in T-cell proliferation and activation. The absence of CXCR3 seems not to interfere with the ex vivo-specific lysis against tumor cells (OT-I CD8 T cells/OVA-expressing tumor cells) nor with the IFNγ, TNFα or Granzyme B expression [11,81]. However, under anti-PD-1 treatment, CXCR3 is required for CD8 T-cell proliferation and activation (IFNγ and TNFα) [81].

### 3.2. CXCR6

CXCR6 is expressed at the cell surface of T cells, mainly the CD4 Th1, CD8 Tc1 and NKT cells, and on NK or cancer cells. Its sole ligand is CXCL16 which is expressed at the cell surface and released as a soluble factor after cleavage by disintegrin or metalloproteinases. CXCL16 is expressed by a wide number of cells within the TME, such as endothelial, mesenchymal stem cells, macrophages, MDSCs, CAFs and cancer cells too [17]. Because of the expression of both CXCR6 and CXCL16 by many cell types, this tandem has a lot of effects on cancer fate. Concerning CD8 T cells, it has been shown that irradiation induces CXCL16 production by breast cancer cells, thus allowing the migration and recruitment of CD8-activated T cells at the tumor site [16]. However, while CXCL16 is highly expressed within pancreatic tumors, CXCR6 is absent from CD8 T cells. Thus, the use of CXCR6-transduced CD8 T cells in pancreatic tumor-bearing mice allows their migration in the tumor where they can play their antitumor role [82]. On the contrary, CXCR6 has been described to be highly expressed in CD8 T cells from murine and human lung and colorectal tumors. CXCR6-deficient CD8 T cells have weaker antitumor effects in murine melanoma, lung and colon cancer models and are less recruited at the tumor site even after anti-PD-1 treatment [83,84]. More than driving the chemoattraction of CD8 T cells, CXCR6 also favors their interaction with DC. Then, the DC-derived IL-15 sustains the effector-like CD8 T-cell survival [85].

### 3.3. CX3CR1

CX3CR1 is expressed at the surface of immune cells, such as T lymphocytes, NK, monocytes, granulocytes or DCs, but also on cancer cells. The ligand of CX3CR1, CX3CL1 (or fractalkine), is expressed on DCs and endothelial and cancer cells [20]. Like CXCL6, CX3CL1 can be cleaved and released by disintegrin or metalloproteinases but also by cathepsin S. Depending on the context, the expression of CX3CR1 and/or CX3CL1, in a wide number of cell types, may lead to opposite effects on cancer progression. Hence, the overexpression of CX3CL1 in tumor cells was shown to inhibit hepatocellular and colon tumor incidence in association with CD4 and CD8 T-cell infiltration in the tumors [86,87]. However, the participation of immune cells in this antitumor effect depends on the localization: in the subcutaneous model, CD8 T cells are responsible for CX3CL1 effects, while in liver localization, it is CD4 T cells [87]. Concerning CD8 T cells, the peripheral blood-circulating CX3CR1^+^ CD8 T-cell frequency is associated with the ICB (anti-PD-1, anti-CTLA-4 or both) efficacy in murine colon cancer models [88]. Similar results were found in a study on a few metastatic melanoma patients under chemo-immunotherapy treatments [89].

### 3.4. CCR2

CCR2 was described to be expressed on many cell types, such as immune and cancer cells. CCL2 (MCP-1) is the ligand with the most important affinity, but CCR2 can also bind CCL7 (MCP-2), CCL8 (MCP-3) and CCL13 (MCP-4) [21]. According to its primary name, CCL2 has the function of a monocyte-chemoattractant protein, but it has been also shown to play a role in CD8 T-cell migration. However, a controversial effect of CCL2/CCR2 on CD8 T cells has been described. For example, the deletion of CCR2 in mice is associated with an increase in the CD8 T cells within the tumors in an N-nitrosomethylbenzylamine (NMBA)-induced esophageal squamous cell carcinoma (ESCC) model. This can be explained by an indirect effect of CCL2 on the recruitment of TAMs (and their differentiation into M2) which inhibit the infiltration of CD8 T cells at the tumor site [90]. In a murine breast cancer model, blocking CCR2 with RS504393 decreases the tumor growth in the presence of anti-PD-1. This combination of treatments does not modify the frequency of CD8 T cells within the tumor, but these cells have a lower expression of exhaustion markers PD-1 and LAG3. The authors also observed a decrease in Treg cells, but the importance of both cells in the antitumor effects of the inhibitor of CCR2 was not investigated [91]. The use of another CCL2 antagonist, *N*-[2-[[(3*R*)−1-[(4-chlorophenyl)methyl]−3-pyrrolidinyl]amino]−2-oxoethyl]−3-(trifluoromethyl)benzamide hydrochloride (BHC) in combination with anti-PD-1 also delays lung tumor apparition, which is associated (but not proved implicated) with an increase in CD8 T cells producing IFNγ [92].

However, the CCL2/CCR2 pathway was described to allow the recruitment of adoptively transferred CD8 T cells within lymphoma murine tumors, and this was associated with tumor growth control [93,94]. Similarly, this pathway was also shown to be involved in the recruitment of type I cytotoxic γδ T cells in melanoma tumors, a hybrid NK/CD8 T lymphocyte able to kill cancer cells, thus conferring an antitumor role of CCL2/CCR2 [95].

One possible explanation for this dual role of CCL2 on CD8 T-cell chemoattraction is post-traductional modifications. Hence, CCL2 has been described to be nitrated/nitrosylated in human colon tumors and in different murine tumor models. This modification inhibits the potential of CCL2 to recruit CD8 T cells in the tumor bed and promote tumor growth. When the production of reactive nitrogen species is inhibited, the CCL2/CCR2 pathway is working to allow CD8 T-cell recruitment and tumor growth control [94].

### 3.5. CCR5

CCR5 is the common receptor of CCL3 (MIP (macrophage inflammatory)-1α), CCL4 (MIP-1β) and CCL5 (RANTES). Both the receptor and ligands are mostly described to be expressed in cancer and immunosuppressive cells (Treg, MDSCs, CAFs and endothelial cells), thus participating in tumor progression [18]. However, it is also expressed on CD8 T cells, thus considering not only to inhibit it but also to activate the recruitment of cytotoxic lymphocytes at the tumor site through this pathway. Hence, the deficiency of CCR5 in mice and more particularly in T cells slows down the tumor growth in different cancer models. Thus, lung cancer cell-derived CCL5 favors the CCR5^+^ CD8 T-cell recruitment and activation, a phenomenon fully achieved by CCR5^+^ CD4 T cells, through the CD40L upregulation and full maturation of APCs [96]. However, an opposing role of CCL5 on CD8 T-cell recruitment was described. In murine renal adenocarcinoma-bearing mice, the use of an anti-CCR5 increases the CD8 T cells (together with CD4 and DCs) within the tumor and retards the tumor progression [97]. Moreover, in murine colon tumor models, the CCL5 deficiency within the cancer cells enhances the CD8 T-cell homing into the tumor, thus leading to the delay of both tumor growth and metastasis. Interestingly, such a deficiency could reinforce anti-PD1 therapy through a perturbation of the TAM metabolism framework [98]. In murine colon cancer models, the CCL5 produced by myeloid cells seems to inhibit CD8 T-cell migration, thus favoring tumor growth [98]. The contradictory results obtained after the inhibition of the CCR5/CCL5 on CD8 T cells and tumor progression might be due to the expression of this receptor/ligand on other cell types, such as immunosuppressive cells, and the proportion of these cells at the tumor site.

Moreover, some chemokines can regulate each other. This is the case for CXCL9 and CCL5. CCL5 regulates the expression of CXCL9 (but not CXCL10) in the TAMs, thus allowing the recruitment of CD8 T cells within the tumor and tumor growth control [9].

Concerning CCL3 and CCL4, it was shown that intratumor basophils are able to secrete these chemokines and to attract CD8 T cells. Thus, expanding these cells with IL-3/anti-IL-3 therapy increases the CD8 T-cell recruitment in melanoma tumors and delays the tumor growth in a CCL3/CCL4-dependent manner [99]. The overexpression of CCL3 in murine colon cancer cells or the subcutaneous injection of CCL3 near the established tumor does not modify the proportion of CD8 T cells but favors their capacity to produce IFNγ. What is interesting in this study is that a low level (500 ng) of CCL3 has an effect, while high amounts (5000 ng) do not, thus suggesting that the effects of chemokines on the recruitment and/or activation of CD8 T cells may partly depend on the quantity produced at the tumor site [100].

### 3.6. CCR6

CCR6 has a unique ligand CCL20 (MIP-3α) which is expressed in many healthy tissues but also by cancer cells and M2 TAMs. CCL20 thus allows the recruitment of CCR6^+^ CD4 immunosuppressive T cells (Th17, Treg) at the tumor site and favors the migration and the metastatic potential of CCR6-expressing cancer cells from diverse types [101]. However, CCR6 was also shown to be expressed by human CD8 T cells [19]. Hence, while human CRC-infiltrating Th17 cells have protumor effects on endothelial cells and neutrophil recruitment through IL-17 and IL-8, respectively, and the presence of these cells within tumors is not correlated with patient survival. This is due to a dual role of Th17 cells and their capacity to secrete CCL20, thus recruiting cytotoxic CD8 T cells [102]. Moreover, CCL20 has an indirect effect on CD8 T-cell recruitment. Indeed, under cisplatin treatment, there is a production of CCL20 and IL-1β by murine lung and breast cancer cells, which triggers the recruitment of the CCR6^+^ type 3 innate lymphoid cells (ILC3) at the tumor site. Then, these cells secrete CXCL10, thus allowing CD8 and CD4 T-cell chemoattraction and improving the ICB efficacy [103].

## 4. Usefulness of Chemokines Released during ICD as a Biomarker

We now know that ICD is mainly driven by two main mechanisms, the antigenicity and the adjuvanticity of dying cancer cells. While the first depends on the exposure of tumor neoantigens (TNAs), tumor-associated antigens (TAAs) and the tumor mutational burden (TMB), the second relies more on an efficient communication between dying cancer cells and immune cells to promote an antitumor immune response.

Focusing on the adjuvanticity of dying cancer cells, it has been recently accepted that chemokine expression, like other immunostimulatory signals such as the extracellular ATP release (find-me signal), calreticulin exposure (eat-me signal) and HMGB1 release, has a key role occurring in this narrow and complex communication between cells which is needed to obtain an efficient recruitment of APCs and to prime a cytotoxic lymphocyte (CTL)-dependent immune response. There is some evidence that the DAMP emission by cancer cells could have a prognostic value in cancer-treated patients. Nevertheless, if a specific chemokine expression could be used as a biomarker of treatment efficacy remains unclear.

### 4.1. As a Negative Prognostic/Predictive Biomarker

CCL5 has been correlated with malignancy, cancer cell proliferation and progressive disease in a number of types of pathologies, such as gynecological, genito-urinary and digestive cancers [104,105,106,107,108]. In node-negative breast carcinoma, the high expression of CCR5 (a CCL5 receptor which may promote tumor progression as mentioned earlier) is an independent prognostic factor of poor survival. In murine models, cancer cells harboring this receptor initiate tumor growth. Reciprocally, the reintroduction of CCR5 into CCR5-negative cells is associated with tumor metastases. An explanation, provided by a single-cell analysis, is that CCR5 may induce the PIK3/Akt pathway leading to cell survival [109]. As expected, CCL5 positivity has an unfavorable prognostic value in stage II breast carcinoma, which is strengthened when combined with estrogen receptor negativity [110]. Similarly, the expression of CCR3 and CCR5 in gastric cancer tissues is also significantly associated with lower survival [111,112]. In this setting, in vitro data suggest that the proliferation of cancer cells harboring CCL5 receptors is related to CD4 T cells, which are the main CCL5 producers among other immune cells. Moreover, a drop in CD8 T cells but not in CD4 T cells is observed when CCL5-treated tumor cells are co-cultured with peripheral blood mononuclear cells (PBMCs). This could indicate some cooperation between tumor and CD4 T cells, resulting in the apoptosis of the cytotoxic population, thus preventing the elimination of the cancer cells [113]. These findings suggest that the CCL5-CCR5 axis potentially has a pejorative prognostic value in several types of malignancies.

Staying aware of the dual role of CCL2, an analysis of a human ESCC cohort confirmed that TAM infiltration was associated with a high level of CCL2, both correlated with a poor outcome. Moreover, the CCL2-CCR2 axis was related to the upregulation of the PD-1 pathway. In CCR2^+/+^ ESCC murine models, most of the TAMs display M2 subtypes, and reciprocally, knocking-out CCR2 dramatically reverses this M2 polarization and enhances the cytotoxic T-cell infiltration. A further investigation shows that tumor escapement from the immune system control is mediated by the PD-L2 expression. Altogether, this suggests that the TAM M2 polarization guided by the CCL2-CCR2 axis leads to immune evasion, going through the PD-L2 immune checkpoint pathway [90]. Some data suggest that CCL2-recruited macrophages also have a promoting role in angiogenesis [114]. In the same way, breast cancer patients displaying a high CCL2 tumor expression and high resident TAM levels were more prone to present early recurrence [115].

Surprisingly, in prostate cancer, after analyzing radical prostatectomy tissues by immunohistochemistry (IHC) and microarray in a multicenter cohort, high CXCL16, CXCR6 and CXCL16-CXCR6 co-expression were found to be independent prognostic factors for poorer clinical failure-free survival (the progression of local symptoms or metastasis to bone, visceral organs or lymph nodes), like other anatomopathological features such as a Gleason > 7, vascular infiltration and positive surgical margins [116].

In colorectal cancer, tumors with a CX3CL1-CX3CR1 negative axis are more prone to have a further tumor relapse or metachronous metastasis [117]. Measuring the chemokine expression in samples of soft tissue sarcoma patients, low mRNA levels of CCL2, CX3CL1 and the combination of both are independent prognostic factors of poor survival. Unexpectedly, this association with a worse outcome is stronger in female than in male patients [118].

The prognostic value of immune marker expression investigated in a cohort of resected pT1 bladder cancer has demonstrated a shorter recurrence-free survival in patients with low mRNA CXCL9 versus a high level. In addition, it is an independent prognostic parameter for decreased overall, disease-specific and recurrence-free survival [119]. Some studies have also shown counterintuitive results. In a prospective study, blood samples were collected from lung cancer patients and healthy controls in order to monitor the plasmatic chemokine levels of CXCL9/10/11 and IFNγ which are known to promulgate T-cell efficacy. There was a higher CXCL9/10 level in the lung cancer patient plasma than the healthy control, and surprisingly, CXCL9/11 were independent prognostic factors for poor survival [120].

### 4.2. As a Positive Prognostic/Predictive Biomarker

Knowing that CD8 presence is correlated with a better outcome, obtaining a successful migration into tumor sites is therefore critical. In order to precisely describe what chemokine is involved in T-cell homing, a transcriptomic approach, conducted in seven types of human cancers, proposed that *CCL5* and *CXCL9* expression is correlated with *CD8A* expression in the tumors [9]. A similar experiment was performed in CRC and found that *CD8A* expression was correlated with high *CCL3, 4, 5, 8, CXCL9*, *10* and *12* expression [121]. Concordant with previous observations, the analyses of metastatic melanoma biopsies showed an association between the presence of CD8 T cells and the expression of a subset of six chemokines (CCL2, 3, 4, 5, CXCL9 and CXCL10). These findings confirm that T-cell homing is mediated by a certain chemokine expression profile [122].

#### 4.2.1. In Localized and Locally Advanced Setting

Chemokine expression could have an interest before and after a treatment with curative intent. Indeed, a low CCL2 level (measured using a cytometric bead array and flow cytometry) in patients’ serum with colorectal cancer before surgery is an independent prognostic factor of better overall survival at 5 years [123]. Interestingly, after surgery in operated colorectal cancer, PCR and IHC analyses were performed on tumor tissues and *CCR5* expression was positively correlated with CD8 T-cell infiltration, whereas the absence or a weak *CCR5* expression correlated with lymph node involvement and with a poorer outcome [124].

The CXCL16 immunohistochemical analysis of 58 colorectal samples, collected during surgery, demonstrated a higher CD4 and CD8 tumor infiltration in patients who exhibited a high level of this chemokine expression. Moreover, a significant longer survival was observed in patients with high CXCL16 levels, suggesting its potential role as a prognostic biomarker, explained by a better improvement in the tumor-infiltrated lymphocytes (TILs) recruitment [125].

The CXCR3-CXCL9/10/11 axis is known to drive the migration of T and NK cells in solid tumors [5]. Concerning CXCL9, a high expression of this chemokine in early breast tumor tissues (RT-PCR or IHC analyses) was associated with a better outcome [126,127]. Focusing on the chemokine receptor CXCR3, its expression on stage III melanoma patients’ peripheral or tumor-involved lymph node CD8 T cells improves survival [128]. A meta-analysis conducted on gastric cancer confirmed the good prognostic value of CXCR3 [129]. An integrative approach using gene expression, phenome mapping, tissue microarrays and the T-cell repertoire in colorectal cancer also exhibited that a high T-cell presence is correlated to the expression of specific adhesion molecules and chemokines (CX3CL1, CXCL10 and CXCL9). Importantly, the high expression of these molecules is translated to a better outcome for patients, confirming that is a benefit conferred by better T-cell homing, mediated by chemokines [130]. As mentioned earlier, other data suggested that the clinical benefit observed with colorectal cancer cells that express CX3CL1-CX3CR1 is more related to the prevention of metastases dissemination than to T-cell infiltration [117]. In other types of cancer, such as early breast carcinoma or gastric adenocarcinoma, high CX3CL1 expression (IHC) is correlated with increased stromal CD8, DC and NK cells and also associated with a disease-free survival benefit for both types of cancer and an overall survival improvement in breast carcinoma [131,132]. Similarly, an overall survival advantage was found in lung adenocarcinoma with an increased CX3CL1 mRNA level [133]. In IDH-1 mutant gliomas, there is a higher NK-κB pathway activation, leading to NK cell migration in a CX3CL1-dependent manner, thus bringing a possible explanation for better outcomes observed in this setting compared to IDH-1 wild-type tumors [134]. Furthermore, in all of these works, CX3CL1 was an independent prognostic factor of better outcomes in each setting [131,132,133,134]. In ESCC tumor tissues (IHC), CXCL10 and CCL5 (also known to be a type 1 DC chemoattractant) were positively associated with local expressions of cytotoxic lymphocyte markers, such as CD8 and granzyme B. Thus, CCL5 expression is positively associated with a better outcome and high CXCL10 is an independent prognostic factor of overall survival and disease-free survival in ESCC [135,136]. Furthermore, for CXCL10-high patients, there was no difference in 5-year overall survival between surgery alone compared to surgery with adjuvant chemotherapy, whereas CXCL10-low patients seemed to benefit from the addition of adjuvant therapy. Consequently, hoping for ulterior validation in prospective studies, CXCL10 expression could serve as a useful marker for the need of adjuvant chemotherapy in locally advanced ESCC [136]. Investigating the role of CXCL11 in gastrointestinal lower tract disease, the transcriptomic approach demonstrated a significant association between high-chemokine expression and longer survival in colon, but not in rectal, adenocarcinoma. A further analysis in a cohort with localized and locally advanced colon cancer confirmed that patients having high CXCL11 in the tumor tissue lived longer. The favorable prognostic value of CXCL11 was confirmed in a multivariable analysis [137]. In the neoadjuvant setting of muscle-invasive bladder cancer, tumor biopsies before treatment revealed that the CXCL11 abundance correlated with high TILs. Moreover, the presence of CXCR3alt-CXCL11 (CXCR3alt resulting from an alternative spliced transcript with more affinity to CXCL11 than CXCL9 or 10) is predictive of the neoadjuvant chemotherapy responsiveness [138].

An mRNA analysis of immune-activating and immunosuppressing factors was conducted in the early breast carcinoma tissue of patients before randomization in the GeparSixto trial, which investigated the impact of adding carboplatin to the neoadjuvant chemotherapy regimen (anthracycline + taxane). Unexpectedly, immunosuppressive (*IDO1*, PD1, PDL1, CTLA4 and *FOXP3*) as well as immune-activating (*CXCL9*, *CCL5*, *CD8A*, *CD80*, *CXCL13*, *IGKC* and *CD21*) markers were both predictors of a pathological complete response, particularly in patients receiving carboplatin, with the highest predictive value for *PDL1* and *CCL5* [139].

#### 4.2.2. In Advanced/Metastatic Setting

Regarding the CXCL9/10/11-CXCR3 axis, a CXCR3 score, reflecting this activation pathway, was created in a cohort of metastatic urothelial carcinoma bladder cancers treated with ICBs and was found to be an independent prognostic factor of good overall survival. There is a higher CXCR3 pathway activation in responders (partial or complete response) than non-responders (stable or progressive disease). In high CXCR3-score patients, there are more ICB responders and consequently longer overall survival. More precisely, there is a higher CXCL9/10-CXCR3-pathway-score protein expression in responder patients. Altogether, this suggests that a higher CXCR3-CXCL9/10 axis activation results in a better response to immunotherapy in this setting [140]. In a French cohort of 60 stage III/IV advanced NSCLC from whom a plasma soluble factors analysis was conducted before an immunotherapy injection, we showed that higher CXCL10 levels were correlated with a better response to first-line treatment, consisting of platinum-based doublet chemotherapy. Moreover, we observed a higher progression-free survival rate in patients with high CXCL10 levels after anti-PD1 therapy. Thus, these data suggest that increased CXCL10 production after receiving chemotherapy is predictive of the ICB further efficacy [39].

In metastatic melanoma patients, biopsies were collected before and post-treatment with ipilimumab, an antibody targeting CTLA-4, and as expected, patients displaying higher immune-related gene expression (including *CXCL9, 10* and *11*) at baseline had a higher total infiltration of lymphocytes and a better response to the ICB. Interestingly, post-treatment biopsy analyses demonstrated an increased expression of a number of genes, including *CXCL11*. Of note, even if this rise in immune gene expression was presented in both responders and non-responders, the amplitude was larger in the responders. Still looking for a clinical benefit, a higher post-treatment expression of *CXCL11* and *CXCR3* was associated with longer survival [80]. In murine models, CXCR3-deficient mice displayed a poor response to anti-PD-1 treatment, and its induction in non-responsive murine tumors restores the ICB efficacy. Moreover, the CXCR3 ligand expression in murine tumors but also in the plasma of melanoma patients is correlated to the ICB antitumor response, highlighting a potential predictive value [81].

Conversely, a weak CCL5-CCR5 axis could be correlated with a better outcome. Indeed, a baseline low serum level of CCL5 in a cohort of metastatic colorectal cancer treated with regorafenib was associated with a survival benefit [141].

Many other chemokines could recruit both anti- and protumor immune cells, and it becomes complex to determine each role of every single chemokine, especially in different organ cancers. Based on this complexity, the prognostic role of *CXCL11* has been explored in a pan-cancer study with 33 types of malignancies. Even though *CXCL11*-high expression correlated with the immune pathway and the presence of CD8 T cells in the TME, its prognostic value nonetheless remains inconsistent across different malignancies [142]. This is why, more than focusing on one chemokine or chemokine receptor, chemokine signatures are in development. A prognosis signature using *CXCL2*, *CXCL13*, *CCL26*, *CCL20* and *CX3CR1* in lung adenocarcinoma turned out to be an independent prognostic factor which could also be predictive of the immunotherapy response [143]. Therefore, a further investigation is needed to ascertain the precise role of chemokines as a prognostic or a predictive biomarkers [144].

## 5. Targeting Chemokine Expression within the Tumor to Improve Treatment Efficacy

The development of immunotherapy, which targets ICBs, has revolutionized the treatment of many solid cancer types. Nevertheless, most patients present an intrinsic resistance to ICBs. Numerous biological phenomena could explain this, such as the loss of MHC-I expression, a low level of tumor neoantigens or weak inflammatory signaling due to poor CD8 T-cell infiltration [145,146,147]. Because of such an intrinsic resistance against ICB monotherapy, new therapeutic tools are required to improve their efficacy. Numerous studies have described the ability of chemotherapies to improve the antitumor immune response by targeting immunosuppressive cells [148] or by inducing immunogenic cell death [149], and a promising way consists of inducing the chemokine expression within the tumor to improve the treatments.

### 5.1. From in Vitro and in Vivo Models toward Clinic

Nowadays, metastatic melanoma requires a 1^st^-line therapy containing ICBs, providing a 5-year overall survival of more than 50% [150]. Nevertheless, some patients experience progressive disease with the poor efficacy of ICBs. Re-examining chemotherapy usefulness, in vitro dacarbazine, temozolomide and cisplatin have the ability to enhance the CCL5, CXCL9 and -10 expression by cancer cells. Moving toward in vivo models, the upregulation of these chemokines after temozolomide treatment is translated into a higher CD3^+^ T-cell homing in a CXCR3-dependent manner. Moreover, using a qPCR in melanoma tumors collected before and after chemotherapy with dacarbazine, a significant increase in *CCL5*, CXCR3 ligands and *CD8* gene expression was observed. Of note, all of these chemokine expression levels were predictors of the tumor response, and patients displaying a high intratumor chemokine level after chemotherapy had a longer survival. Interestingly, CCL5, which has been mainly correlated with cancer progression, seems to be synergic with CXCR3 ligands. All of these findings suggest that the benefit in chemotherapy-sensitive tumors could be mainly driven by the antitumor immune response, opening the door to a potential combotherapy in order to improve the ICB efficacy [59]. The capacity of regorafenib to increase the CD8 T-cell recruitment in murine hepatocellular carcinoma models was also observed in humans. In blood samples from patients treated with regorafenib, the CXCL10 concentration increases along with the treatment which confirms this chemokine induction in humans [70].

Using single-cell RNA sequencing on biopsies from murine CT26 colon cancer tumors before treatment, Qu et al. identified a macrophage resident population correlated with the response to avelumab (anti-PD-L1). This TAM pro-inflammatory subset displayed a high CXCL9 expression profile, thus attracting CXCR3^+^ effector cells in the tumor. Regarding human cohorts treated with either avelumab or atezolizumab, it appeared that a CXCL9-high expression was associated with longer overall survival. These data highlight the existence of the TAM subpopulation harboring a pro-inflammatory chemokine signature, thus leading to a better response to ICBs. Finding a way to promote CXCL9 production in a tumor microenvironment appears interesting [58]. 

### 5.2. In Early Phase Trials

There is emerging evidence in early phase trials that chemokine expression improves the outcome. In a phase I/II trial with a stage IV or unresectable stage III advanced melanoma treated with TEBENTAFUSP-a TCR/anti-CD3 bispecific fusion protein targeting a TAA in melanoma-increasing CXCL10 expression correlates with the reduction in peripheral CXCR3^+^ CD8 T cells and with at least a 2-fold increase in the number of intratumor T cells after treatment (the pre- and post-treatment tumor biopsy IHC showing a greater presence of T-cell markers after treatment). Moreover, a greater increase in the CXCL10 serum level is associated with longer overall survival and better tumor shrinkage. Similar effects are observed with a greater increase in CXCL11. Consistently, a greater decrease in the circulating CXCR3^+^ CD8 T cells is also associated with a better outcome. What is really relevant for clinicians is that the rash occurring in treatment correlates with longer overall survival, a greater-fold increase in the CXCL10 serum level and a decrease in the circulating CXCR3^+^ CD8 T cells, showing that this eruption testifies to the treatment efficacy by redirecting the CD8 T cells into tumors [151].

Using DCs transduced with an adenoviral vector expressing the CCL21 gene (a ligand of CXCR3 and CCR7), an autologous intratumor injection, guided by bronchoscopy or CT-scan, was performed in a few patients harboring stage III/IVB NSCLC. The TAA-specific peripheral blood lymphocyte induction of IFNγ demonstrated a systemic immune response in nearly 38% of patients. In analyzing tumor biopsies, more than half of the cohort displayed a 3-fold average increase in tumor-infiltrated T cells. Interestingly, patients for whom vaccination resulted in a higher CD8 infiltration showed a greater PD-L1 mRNA expression, suggesting negative feedback of the immune response was elicited with the vaccine. These observations suggest that in situ vaccination with DC-CCL21 promotes a systemic and specific antitumor immune response which might be synergic with immunotherapy [152].

A new immunotherapy approach using Coxsackie virus A21 (CVA21), which targets cells expressing the intercellular adhesion molecule-1 (ICAM-1), was explored in non-muscle-invasive bladder cancer patients after promising in vitro results. Patients received either CVA21 alone or with a mitomycin C low dose, a chemotherapy which previously provided evidence of the ICAM-1 upregulation on cancer cells. Each regimen demonstrated an increased inflammation in the tissue biopsies. The transcriptomic analysis comparing the treated and non-treated patients illustrated a significant upregulation of a number of genes correlated with the IFNγ and Th1 immune response, such as *CXCL10* and *CXCL11* in treated patients’ tissues. Withal, there was a concomitant boost of the immune checkpoint genes *CD274* (PD-L1), *CD273* (PD-L2) and *LAG3*, suggesting again a negative feedback initiation. From a clinical point of view, no safety issues were reported. Such data pointed out the ability of this virus-mediated therapy to heat the immunologic tumor microenvironment, also suggesting that a combination with ICBs would be interesting to explore [153].

In the setting of recurrent platinum-sensitive ovarian cancer, a phase I trial combining intraperitoneal chemotherapy, TLR3 ligand, IFNα and an oral COX-2 blocker raised the local chemokine expression, such as CXCL9, 10 and 11, which are known for their lymphocyte-attractive role. This chemokine-modulatory regimen (CKM) also triggers the upregulation of MHC I/II, perforin and granzymes, testifying to TIL activity. Adding a tumor-loaded dendritic cell vaccine to such therapy will be further under investigation [154].

## 6. Conclusions

In this review, we have seen that the study of chemokines and more particularly chemokines induced by treatments, such as ICD inducers, is very difficult. Actually, one chemokine may attract different cell types expressing its specific receptor with a different effect on the antitumor response. For example, the presence of CXCL10 or CXCL11 within the tumor is associated with a CD8 T-cell number but also with the CD4 Treg cell accumulation with immunosuppressive and cancer-initiating effects, thus rendering it difficult to use such chemokines as prognostic factors [155,156]. Beyond the single effect of immune cell attraction, it is important to remind that the expression of different chemokines, either by immune cells or tumor cells, has a role in shaping the tumor microenvironment framework in different ways: while the Th1 cell signature provides a good prognostic, the orientation toward Th2, Th17 and Treg cells yields a worse one [157]. For example, apart from chemoattraction, chemokines can also affect other cellular processes, such as T-cell differentiation (CCL3/CCR5-Th1 or CCL2/CCR2-Th2) or APC functions (CCL3, 4, 5/CCR5 and CCL2/CCR2-IL-12 production), macrophage polarization (CXCR3-M2) or cancer cell metastasis (CXCL10, CCL5, CCL2) [108,158,159,160,161,162].

Rising in complexity, one chemokine may have different effects according to the quantity produced: while high CCL3 or CXCL12 have chemorepulsive effects, low CCL3 or CXCL12 have chemoattractive effects toward CD8 T cells. [100,163]. Such an ambivalent effect was also observed for the IFNγ a factor involved in chemokine expression [164].

All these arguments make the possibility of using chemokines as biomarkers or therapeutic targets difficult. However, based on the works exposed in this review, some chemokines seem to be relevant markers of CD8 T-cell recruitment in the tumor and predictive companion markers of patients’ responses. Moreover, the preclinical development of new therapeutic tools is promising, such as chemokine inductors or virus-mediated chemokine delivery. A number of early phase trials focusing on chemokine modulation to treat cancer are ongoing, with a few of them already completed with available results (Table 2).

Finally, even if all the studies did not always link the chemokine production at the tumor site with ICD, the use of chemotherapy and/or radiation able to induce such a cell death argues for the participation of the ICD-mediated chemokine production in CD8 T-cell chemoattraction and in tumor growth control in some specific contexts.

## Figures and Tables

**Table 1:** Main receptor/chemokine described to participate in CD8 T-cell migration.

Chemokine Receptors	Chemokines	Ref
CXCR3	CXCL9, 10 and 11, CCL21	[7,8,9,10,11,12,13]
CXCR4	CXCL12	[14,15]
CXCR6	CXCL16	[16,17]
CCR5	CCL3, CCL4, CCL5	[10,18]
CCR6	CCL20	[19]
CX3CR1	CX3CL1	[20]
CCR2	CCL2	[21]

**Table 2:** Early phase trials focusing on chemokine modulation to treat cancer.

Target	NCT	Phase	Drugs	Type of Cancer	Associated Treatments
Chemokine-modulatory regimen (CKM) in order to modulate cancer immune response	NCT01545141 NCT03403634	I/II	Celecoxib IFN-α2b Rintatolimod (TLR3 agonist)	Colorectal	None
	NCT03599453	I		TNBC	+ Pembrolizumab
	NCT03899987	II		Prostate	Aspirin instead of celecoxib
	NCT02151448	I/II		Peritoneal	+ αDC1 vaccine
	NCT04093323	II		Melanoma	+ αDC1 vaccine + anti-PD-1/PD-L1
CCL21	NCT01433172	I/II	CD40L-expressing bystander cell line GM.CD40L vaccine + CCL21	Lung adenocarcinoma	None
CCL2	NCT00992186	II	Anti-CCL2 mAb	mCRPC	None
	NCT00537368	I		Solid tumors	None
CCR2	NCT02732938	II	CCR2 antagonist PF-04136309	Pancreas	Gemcitabine, Nab-paclitaxel
CCR5	NCT04504942	II	Anti-CCR5 mAb	Solid tumors	None
	NCT03838367	I/II		TNBC	Carboplatin
	NCT03631407	II	Vicriviroc	Colorectal	Pembrolizumab
	NCT01276236	II	Maraviroc	Kaposi’s sarcoma	None
CXCR4	NCT02737072	I	CXCR4 peptide antagonist LY2510924	Solid tumors	Durvalumab
	NCT05465590	I	Anti-CXCR4 peptide–drug conjugate MB1707 (PDC)	Solid tumors	Paclitaxel-conjugated
	NCT04543071	II	CXCR4 antagonist motixafortide	Pancreas	Cemiplimab, Gemcitabine, Nab-paclitaxel
	NCT04177810	II	CXRC4 antagonist plexirafor	Pancreas	None
CXCL12	NCT03168139	I/II	CXCL12 pegylated L-oligoribonucleotide antagonist (olapsted pegol NOX A-12)	Pancreas, colorectal	Pembrolizumab

mCRPC: metastatic castrate-resistant prostate cancer; TNBC: triple-negative breast cancer.
